# Human inborn errors of immunity associated with IRF4

**DOI:** 10.3389/fimmu.2023.1236889

**Published:** 2023-09-22

**Authors:** Romane Thouenon, Sven Kracker

**Affiliations:** ^1^ Université Paris Cité, Paris, France; ^2^ Laboratory of Human Lymphohematopoiesis, Imagine Institute, INSERM UMR, Paris, France

**Keywords:** interferon regulatory factor family, IRF4, inborn errors of immunity, cancer, primary antibody deficiency

## Abstract

The transcription factor interferon regulatory factor 4 (IRF4) belongs to the IRF family and has several important functions for the adaptive immune response. Mutations affecting IRF family members IRF1, IRF3, IRF7, IRF8, or IRF9 have been described in patients presenting with inborn errors of immunity (IEI) highlighting the importance of these factors for the cellular host defense against mycobacterial and/or viral infections. IRF4 deficiency and haploinsufficiency have been associated with IEI. More recently, two novel IRF4 disease-causing mechanisms have been described due to the characterization of IEI patients presenting with cellular immunodeficiency associated with agammaglobulinemia. Here, we review the phenotypes and physiopathological mechanisms underlying IEI of IRF family members and, in particular, IRF4.

## Introduction

The interferon regulatory factor (IRF) consists of a family of nine human transcription factors (1, 2). IRF1 was the first protein of this family identified (3) and was shown to induce the two type I interferons (IFNs) IFNα and IFNβ (4, 5). It was shown that IRF1 regulates a variety of target genes. Although both type I and type II IFNs induce IRF1 expression, IRF1-dependent responses to IFN-γ appear to be much stronger than those to type I IFNs. Subsequently, other proteins were associated with this family because of their structural and functional (as transcription factors) similarities (**Supplementary Figure 1**) (1, 6–9). The unique function of a particular IRF is related to a combination of cell-type-specific expression and interactions with other members of the IRF family and/or other transcription factors (8). They are expressed in different tissues with IRF1, IRF2, IRF3, and IRF6 present in almost every tissue; IRF4 and IRF8 mainly in hematopoietic cells; IRF5 in bone marrow, spleen, and lymph nodes; IRF7 in liver and spleen; and finally IRF9 in female genitals and in the respiratory tract. On the cellular level, specifically in hematopoietic cells, IRF1 and IRF2 are mainly expressed in neutrophils and monocytes. IRF3 expression is detected in all hematopoietic lineages with high expression in B and T lymphocytes. IRF4 is expressed in B and T lymphocytes as well as in dendritic cells (DCs). IRF5 is expressed in monocytes, B cells, and DCs. IRF6 expression is low in immune cells. IRF7 and IRF8 are expressed in DCs, and IRF8 expression is also detected in B cells and monocytes. IRF9 is expressed in all hematopoietic lineages with high expression in granulocytes (the human protein atlas https://www.proteinatlas.org/). All IRFs are regulated by IFNs, except IRF4 (10), whose expression is controlled by antigen receptors, Toll-like receptors, and CD40 signaling pathways (11, 12). IRF proteins contain two functional domains: an N-terminal helix-turn-helix DNA-binding domain (DBD) containing conserved tryptophan residues, and a C-terminal interferon activation domain (IAD, also known as the IRF-association domain) known to mediate protein–protein interactions (13–15). Phylogenetic analysis of IRF DBD indicated that human IRF can be divided into four subfamilies: the IRF1 subfamily (including IRF1 and IRF2), the IRF3 subfamily (including IRF3 and IRF7), the IRF4 subfamily (including IRF4, IRF8, and IRF9) and the IRF5 subfamily (including IRF5 and IRF6) (15). IRF4 contains a C-terminal flexible autoinhibitory region that binds directly to the DBD to modulate its interaction with DNA, in contrast to the other IRF4 subfamily members (**Supplementary Figure 1**) (13). IRF family members are known to be important for immune cell development and functions. All IRF proteins stimulate the expression of IFN-inducible genes (16–18), except IRF2 that antagonizes IRF1, hence attenuating type I IFN response (6, 19). IRF1, IRF3, IRF5, and IRF7 also enhance TLR-dependent gene induction (20–24) and IRF8 stimulates IFN-γ- and PAMP-inducible genes (25, 26). IRF4 regulation differs from that of the other members of the IRF family and the *IRF4* gene has been described as a sensor of B-cell receptor or T-cell receptor signaling (27, 28). In response to BCR/TCR signaling, the *IRF4* gene is expressed through the NFκB signaling pathway. Subsequently, signaling-induced changes in the IRF4 protein level control the fate of activated B and T lymphocytes (27–29).

## IRF family member variants associated with cancer

**Figure 1:**
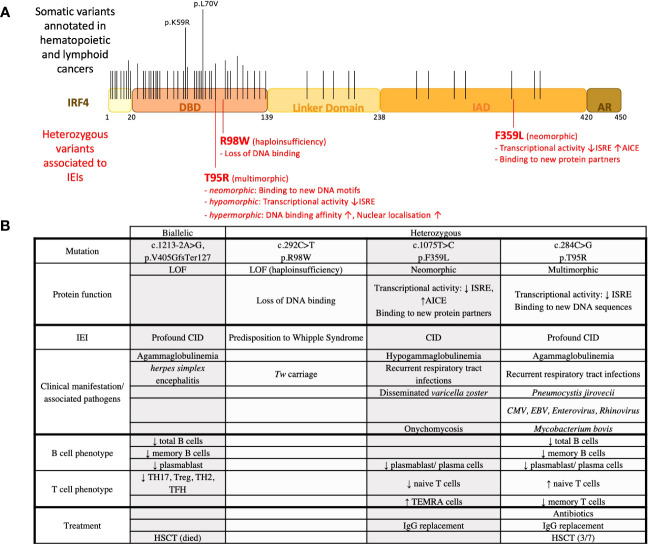
IRF4 variants associated with IEIs and observed in hematopoietic cancers. **(A)** IRF4 variants found in IEIs and hematopoietic cancers. Black lines indicate the location of recurrent somatic mutations noted in COSMICv97. Long lines indicate mutations that were found more frequently. Red lines indicate the location of heterozygous variants found in patients with IEI. **(B)** IRF4 variants associated with IEI. Clinical and immunological phenotypes of patients are recapitulated.

The IRF protein family members were also found in the literature to be associated with different kinds of cancer. For example, IRF1, IRF2, IRF5, IRF8, and IRF9 were found to be upregulated in glioma, and their mRNA levels correlated to the tumor grade and the clinical outcome of the disease (30). Moreover, IRF1 was identified to be deleted in patients presenting with leukemia, myelodysplastic syndromes for hematopoietic malignancies (31), and esophageal and gastric cancers (32, 33). The pathophysiological role of IRF2 in cancer was mainly associated with impaired immune surveillance associated with IFN-mediated CD8 T-cell exhaustion (34). Furthermore, in colorectal cancer, loss of IRF2 expression mediated by oncogenic KRAS promotes the migration of myeloid-derived suppressor cells to the tumor microenvironment driving resistance to anti-PD-1 therapy (35). IRF8 has also been associated with the induction of T-cell exhaustion in cancer microenvironment (36, 37). IRF3 expression was linked to a better survival outcome mainly for gastrointestinal cancers (38, 39). IRF4 chromosomal translocation/mutation was mainly associated with hematopoietic cancers and found in particular in multiple myeloma (40), large B-cell lymphoma (41), and adult T-cell lymphoma-leukemia (42). The catalog of somatic mutation in cancer (COSMIC v97) contains 146 somatic missense variants identified in hematopoietic cancers affecting the whole IRF4 gene (**Figure 1A**). Of note, the two most frequent IRF4 mutations annotated in COSMIC v97 p.K59R (c.176A>G) and pL70V (c.208C>G) were located in IRF4’s DBD (42).

## IRF family member variants associated with IEI

IEI affecting IRF family members present with defective control of viral infection and/or mycobacterial infections (**Table 1**). IRF1 autosomal recessive (AR) loss of function (LOF) variants have been identified in two related patients presenting with Mendelian susceptibility to mycobacterial diseases (MSMD). Functional characterization showed, in addition to a defect of NK, naive αβ CD8+ T and myeloid DC differentiation, the absence of IRF1 impacting IFN type II (IFNγ) production, leading to the development of MSMD (43). IRF3 autosomal dominant (AD) LOF variants have been described for a patient presenting with HSV-1 encephalitis and for another patient presenting with recurrent cutaneous herpes. This has been characterized as an impairment of the type I IFN expression after HSV-1 infection in these patients (44–47). AR LOF IRF7 variants were also found in patients presenting with severe infection by influenza virus and SARS-CoV-2-induced life-threatening induced pneumonia. The impairment of IFN type I and type III expression was observed in plasmacytoid DCs (pDCs) deficient in IRF7 and has been associated with poor clinical outcome (47, 52–54). Then, AD and AR LOF variants found in the IRF8 gene were also found in patients presenting with MSMD (55, 56, 60). AD IRF8-deficiency caused impaired development of conventional DC and Th1 cells, whereas AR IRF8 deficiency was associated with an absence of circulating monocytes and DCs, and with reduced NK cell numbers and function in some patients (55, 56, 60). IRF9 AR LOF variants were found in patients presenting with severe influenza virus infection and might result in susceptibility to infection by a wide range of viruses. Functionally, it has been associated with compromised type I and III IFN signaling, which then leads to defective control of viral infections (57–59). Finally, variants found in the *IRF4* gene have also been associated with IEI, and this aspect will be developed in the following section.

**Table 1:** Variants affecting IRF family members found in IEI patients.

Gene	Inheritance	Allele	IEI and infectious phenotype	References
IRF1	AR	LOF	Mendelian susceptibility to mycobacterial diseases (MSMD)	(43)
IRF3	AD	LOF	Herpes simplex encephalitis (HSE)	(44)
IRF3	AD	LOF	Critical influenza and COVID-19 pneumonia	(45–47)
IRF3	AD		Recurrent HSV-1 cutaneous infections	(45)
IRF4	AD	Multimorphic	CID	(48)
IRF4	AD	Neomorphic	CID	(49)
IRF4	Uniparental isodisomy	LOF	CID	(50)
IRF4	AD	LOF	Whipple syndrome	(51)
IRF7	AR	LOF	Severe influenza	(52–54)
IRF7	AR/AD	LOF	COVID-19 pneumonia	(47, 53)
IRF8	AD	LOF	Milder form of mendelian susceptibility to mycobacterial diseases (MSMD)	(55, 56)
IRF8	AR	LOF	Mendelian susceptibility to mycobacterial diseases (MSMD)	(56)
IRF9	AR	LOF	Susceptibility to infection by a wide range of viruses, including severe influenza	(57–59)

AD, autosomal dominant; AR, autosomal recessive; LOF, loss-of-function; CID, combined immunodeficiency.

## IRF4 variants associated with IEI

### IRF4 complete deficiency due to uniparental isodisomy

The first IRF4 LOF variant associated with IEI reported in the literature was a case report of an IRF4 deficiency inherited by uniparental isodisomy found in a patient with profound combined immunodeficiency (50). The patient died at 2 years of age shortly after an allogeneic hematopoietic stem cell transplantation. Immunological evaluation revealed an agammaglobulinemia with an absence of memory B cells and plasma cells (in blood, bone marrow, and lymph nodes). Only a small percentage of regulatory CD4 T cells was observed and CD8 effector memory T cells were undetectable. Histological analysis of lymph node biopsies showed a loss of normal architecture characterized by a lack of follicles and the presence of necrotic areas. An increased number of pDCs was noted whereas an inadequate differentiation of monocytes towards monocyte-derived DCs was observed *in vitro* (based on the lack of CD1a expression). Genetic analysis identified a mutation, c.1213-2A>G, p.V405GfsTer127, in the IRF4 gene located on chromosome 6 found to be homozygous in the patient and heterozygous in the mother (**Figure 1B**). Results of a single-nucleotide polymorphism array suggested a maternal uniparental isodisomy of chromosome 6 (50). Because of the very complex genetics of this unique case of IRF4 deficiency, the possible involvement of other genetic factors cannot be excluded.

### IRF4 haploinsufficiency associated with Whipple’s disease with incomplete penetrance

Whipple’s disease (WD) is an intestinal inflammatory disease due to infection by the bacteria *Tropheryma whipplei* (*Tw*). The vast majority of infected individuals clear *Tw* primary infections; however, a minority become chronic asymptomatic carriers, of whom a very small proportion develop WD (61, 62). IRF4 haploinsufficiency associated with WD has been reported due to the study of four related patients diagnosed with WD with a mean age of diagnosis of 58 years (51). The four patients from a large multiplex family were found carriers of a heterozygous missense IRF4 variant (c.292 C>T, p.R98W). Eight other relatives were heterozygous for this variant. Interestingly, five of them were reported to be *Tw* carriers with a mean age of 55 years. The familial segregation of the IRF4^R98W^ allele was therefore consistent with an autosomal dominant pattern of WD inheritance with an age-dependent incomplete penetrance. Of note, chronic *Tw* carriage also followed an autosomal dominant pattern. Immunological abnormalities were not reported. However, it is unclear to which extent in-depth immunophenotyping was performed for symptomatic and asymptomatic IRF4^R98W^ allele carriers. Functional characterization of the IRF4^R98W^ variant showed that this IRF4 variant has lost its ability to bind to known IRF4 DNA motifs like interferon-stimulated response elements (ISREs). In addition, a higher level of IRF4 protein in the cytoplasm was observed in cells from IRF4 R98W allele carriers than in wild-type homozygotes. Thus, as a mechanism, haploinsufficiency not due to loss of expression but due to lack of activity of the IRF4^R98W^ protein variant in the nucleus has been proposed (51) (**Figures 1A, B**). Whether IRF4 has a function in the cytoplasm and whether IRF4^R98W^ protein interferes with it remain to be elucidated.

### IRF4 autosomal dominant variant with neomorphic functions

A heterozygous IRF4 variant with neomorphic functions has been discovered due to the study of three patients from a multigenerational non-consanguineous family. All three patients suffered from recurrent respiratory tract infections associated with hypogammaglobulinemia and reduced circulating plasmablasts/plasma cells. The patients presented with early (teenage-onset) hair graying and skin depigmentation. This is possibly associated with the reported IRF4 expression in melanocytes and its role in the MITF/Tyr signaling pathway (63). Of note, IRF4 single-nucleotide polymorphisms (SNPs) have been associated with a light skin and hair color (49). For the T-cell compartment, they presented with low naïve T-cell counts and high effector memory counts for both CD4 and CD8 T cells. Genetic analysis of these patients revealed a germline heterozygous missense *IRF4* gene variant (c.1075 T>C, p.F359L) affecting the IRF4 protein’s IAD (64). IRF4 is known to bind to erythroblast-transformation-specific (ETS) interferon composite elements (EICEs) as an PU-1/IRF4 heterodimer and to AP-1-IRF composite elements (AICEs) as an AP-1 (composed of BATF and JUN)/IRF4 heterodimer (27, 65, 66). At high nuclear protein level, IRF4 is known to bind to ISRE, allowing the formation of the IRF4 homodimer (14) and the expression of pro-plasmablast genes as *XBP1* and *PRDM1* (67). A combination of different functional analysis including luciferase reporter assays, chromatin immunoprecipitation sequencing, and transcriptomic analysis indicated that the IRF4^F359L^ variant deregulated IRF4 transcriptional activity on AICE and ISRE sites. On AICE sites, an increased transcriptional activity mediated by the IRF4^F359L^ variant was observed. In contrast, the IRF4^F359L^ variant supressed transcription on ISRE sites. Proteomic analysis discovered the transcriptional repressor ETV6 as a novel protein binding partner for IRF4^F359L^. Notably, ETV6 has already been shown to be a negative regulator of IRF8 transcriptional activity on ISRE sequences (68). As the transcriptional activity mediated by IRF4^F359L^ on ISRE sites in ETV6-knockdown cells was comparable to the one of IRF4 wild-type expressing cells, it is reasonable to suggest that, on ISRE sites, the presence of IRF4^F359L^ allows the recruitment of ETV6, resulting in the suppression of IRF4-triggered transcriptional activity. One other protein interacting with IRF4^F359L^ is JUN-B. The family of JUN proteins is composed of c-JUN, JUN-B, and JUN-D, and they differ in their binding affinity to AP-1 (69). For T-cell differentiation, JUN-B has been mainly associated with the generation of regulatory T cell and Th17 cells (70–72). Thus, the high terminal effector counts for CD4 and CD8 T cells could be associated with an increased activity of IRF4^F359L^ (possibly in conjunction with JUN-B) on AICE sequence. Of note, IRF4^F359L^ appears to function in heterochromatin organization as evidenced by diffuse nuclear H3K9 trimethylation immunofluorescent signals in patients’ derived B-EBV cells. This might be associated with a loss of IRF4^F359L^ binding to PC4 (64), a complex component reported to function in heterochromatin organization in activated B lymphocytes (48, 73) (**Figures 1A, B**). Taken together, deregulation of IRF4 transcriptional activity causing an AD IEI can be associated with new protein interaction(s) mediated by an IRF4 neomorphic variant.

### IRF4 autosomal dominant variant with multimorphic functions

Seven patients were found around the world all presenting with early-onset (<1 year of age) recurrent sinopulmonary infections associated with agammaglobulinemia and a lack of circulating plasma cells even though circulating B cells were found (74). Six out of the seven patients suffered from *Pneumocystis jirovecii*, causing pneumonia. In addition, clinical complications associated with *Mycobacterium bovis*, cytomegalovirus, and EBV infection were reported for some of the patients. For both CD4 and CD8 T cells, patients presented with high naive counts associated with low central, effector, or terminal effector memory cells. Genetic analysis of these patients led to the identification of the very same heterozygous germline mutation affecting the DBD of the IRF4 protein (c.284C>G, p.T95R). Functional analysis showed that transcriptional activity of the IRF4^T95R^ was largely reduced on ISRE sites. This low transcription activity on ISRE sites was functionally associated with a failure of patients’ B cells to differentiate into plasmablast/plasma cells and to secrete Ig. The generation of class-switched and memory B cells was reduced. T cells from the patients failed to differentiate into TH17 and TFH cells and showed a reduced IL-2 and IFNγ secretion. Knock-in mice with the identical T95R substitution recapitulated the immunodeficient phenotype observed in these patients. Interestingly, *Irf4* ^T95R^ knock-in mice indicated an expansion of nonspecific germinal center B cells at the expense of antigen-specific germinal center B cells. Consistent with an increased affinity for DNA, the subcellular localization of IRF4^T95R^ was altered, with an increased nuclear-to-cytoplasmic ratio compared with IRF4 wild type (74). CHIP-Seq analysis performed on patients’ derived B-EBV cells in conjunction with a deep learning tool (ExplaiNN) (75) unveiled IRF4^T95R^-altered DNA binding specificity. ExplaiNN identified a noncanonical GATA-containing ISRE motif and various noncanonical AICE motifs that appeared important for IRF4^T95R^ binding but detrimental to IRF4 WT-specific binding. This altered DNA binding capacity of IRF4^T95R^ can have a functional impact on the expression of new target genes containing these non-canonical AICE sites, as shown for CXCL13 (74). As such, IRF4^T95R^ presents with a simultaneous multimorphic combination of loss, gain, and new functions for IRF4. Three out of the seven patients have undergone allogenic hematopoietic stem cell transplantation; however, reported follow-up time was less than 8 months for all them. Of note, the T95R variant has been identified as somatic mutation in adult T-cell leukemia implicating multimorphic IRF4 function also in lymphomagenesis (**Figures 1A, B**). Additional somatic mutation affecting the IRF4 DBD was already associated with lymphomagenesis, possibly suggesting that other germline variants affecting IRF4’s DBD might also cause IEI. Whether these IRF4 DBD variants present with a multimorphic IRF4 pathophysiology as described for IRF4^T95R^ remains to be determined.

## Conclusion

Genetic characterization of IEI is essential for an exact diagnosis and accurate treatment of patients. The use of next-generation sequencing for genetic exploration/diagnosis of IEI facilitates the discovery of novel genetic lesions (either heterozygous or biallelic) underlying IEI. Moreover, the understanding of pathophysiological mechanisms underlying IEI is evolving. Several genetic lesions in transcription factors of the IRF family are associated with IEIs, and within this family, *IRF4* mutations can cause IEIs both at the heterozygous and biallelic state. Autosomal dominant *IRF4* gene-associated pathophysiological mechanisms include haploinsufficiency, neomorphic, and multimorphic (a simultaneous combination of loss, gain, and new functions for IRF4), showcasing the complexity underlying IRF4 deregulation. Studying the pathophysiology of autosomal dominant IRF4 protein variants and their impact on transcriptome, epigenome, and chromatin structure organization will provide important new insights into IRF4 function and might have implications for treatments of IEI and possibly even cancer. In conclusion, the continuously increasing genetic delineation of IEI, besides its essential role in clinics, allows the description of new unsuspected physiological mechanisms, enriching our knowledge on immune responses.

## Author contributions

RT and SK contributed to conception and design of the study. RT wrote the first draft of the manuscript. SK wrote sections of the manuscript. All authors contributed to manuscript revision, read, and approved the submitted version.

## Supplementary material

Supplementary Figure 1 Comparison of IRF family protein structure. Schematic representation of the protein structure with indication of the DNA binding domain (DBD), the linker domain, the interferon association domain (IAD), and the autoinhibitory region.
