# Supplementary figures and images for: Human inborn errors of immunity associated with IRF4
Source: Front Immunol. 2023 Sep 22;14:1236889. doi: 10.3389/fimmu.2023.1236889 (PMC10556498; doi:10.3389/fimmu.2023.1236889)

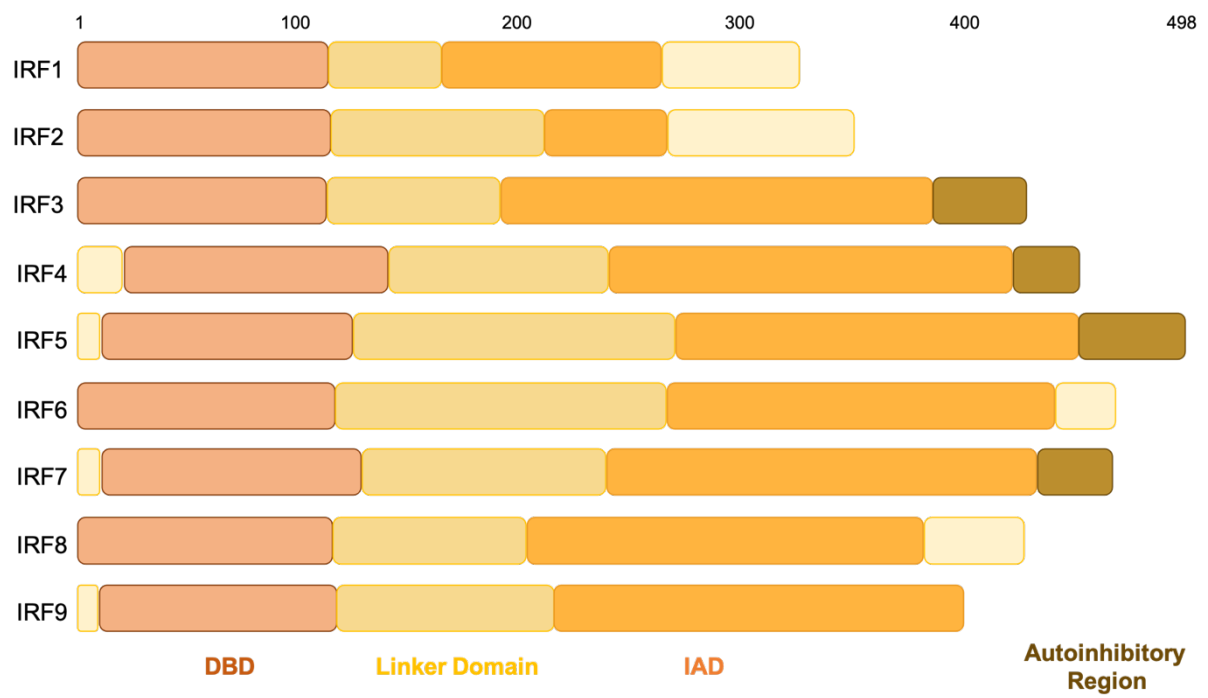

Figure 1: Comparison of IRF family protein structure

Supplement: Supplementary Figure 1 — Comparison of IRF family protein structure. Schematic representation of the protein structure with indication of the DNA binding domain (DBD), the linker domain, the interferon association domain (IAD), and the autoinhibitory region. [file DataSheet_1.pdf]
